# Biventricular Tissue Tracking with Cardiovascular Magnetic Resonance: Reference Values of Left- and Right-Ventricular Strain

**DOI:** 10.3390/diagnostics13182912

**Published:** 2023-09-11

**Authors:** Andrea Barison, Roberto Ceolin, Alessandro Palmieri, Pietro Paolo Tamborrino, Giancarlo Todiere, Chrysanthos Grigoratos, Ignazio Alessio Gueli, Carmelo De Gori, Alberto Clemente, Laura Pistoia, Alessia Pepe, Giovanni Donato Aquaro, Vincenzo Positano, Michele Emdin, Filippo Cademartiri, Antonella Meloni

**Affiliations:** 1 Cardiology and Cardiovascular Medicine, Fondazione Toscana Gabriele Monasterio, 56124 Pisa, Italy; 2 Institute of Life Sciences, Scuola Superiore Sant’Anna, 56127 Pisa, Italy; 3 Cardiothoracovascular Department, Azienda Sanitaria Universitaria Giuliano Isontina (ASUGI), University of Trieste, 34128 Trieste, Italy; 4 Cardiothoracovascular Department, Careggi University Hospital, 50134 Florence, Italy; 5 Cardiology Division, Cardiothoracic and Vascular Department, Pisa University Hospital, 56124 Pisa, Italy; 6 Department of Radiology, Fondazione Toscana Gabriele Monasterio, 56124 Pisa, Italy; 7 Clinical Research Unit, Fondazione Toscana Gabriele Monasterio, 56124 Pisa, Italy; 8 Institute of Radiology, Department of Medicine, University of Padua, 35128 Padova, Italy; 9 Academic Radiology Unit, Department of Surgical Medical and Molecular Pathology and Critical Area, University of Pisa, 56124 Pisa, Italy; 10 Department of Bioengineering, Fondazione Toscana Gabriele Monasterio, 56124 Pisa, Italy

**Keywords:** magnetic resonance imaging, strain, cine, tissue tracking, systolic function, myocardial deformation

## Abstract

We derived reference values of left-ventricular (LV) and right-ventricular (RV) strain parameters in a cohort of 100 healthy subjects by feature tracking cardiac magnetic resonance (FT-CMR). Global and regional strain values were calculated for the LV; circumferential and radial_SAX_ strain parameters were derived from the short-axis (SAX) stack, while longitudinal and radial_LAX_ strain parameters were assessed in three long-axis (LAX) views. Only global longitudinal strain (GLS) was calculated for the RV. Peak global LV circumferential strain was −16.7% ± 2.1%, LV radial_SAX_ strain was 26.4% ± 5.1%, LV radial_LAX_ strain was 31.1% ± 5.2%, LV GLS was −17.7% ± 1.9%, and RV GLS was −23.9% ± 4.1%. Women presented higher global LV and RV strain values than men; all strain values presented a weak relationship with body surface area, while there was no association with age or heart rate. A significant association was detected between all LV global strain measures and LV ejection fraction, while RV GLS was correlated to RV end-diastolic volume. The intra- and inter-operator reproducibility was good for all global strain measures. In the regional analysis, circumferential and radial strain values resulted higher at the apical level, while longitudinal strain values were higher at the basal level. The assessment of cardiac deformation by FT-CMR is feasible and reproducible and gender-specific reference values should be used.

## 1. Introduction

The assessment of myocardial deformation is of utmost importance for the early diagnosis, staging, and follow-up of almost all cardiovascular diseases. In addition to the established role of left-ventricular (LV) and right-ventricular (RV) ejection fraction as a global index of systolic function, myocardial strain has emerged as a more sensitive parameter to assess biventricular function [1]. Transthoracic echocardiography represents the most widespread imaging tool for the assessment of biventricular systolic and diastolic function of both ventricles, including tissue Doppler imaging and deformation imaging (strain and strain rate) [2], but cardiac magnetic resonance (CMR), with its inherently high spatial resolution, superior signal-to-noise ratio, and multi-parametric and tomographic nature, represents an attractive imaging modality that is uniquely able to provide morphology, function, perfusion, viability, and tissue characterization all in a single examination [3]. Several CMR sequences have been specifically developed to assess myocardial deformation, including tissue-tagging CMR, tissue phase mapping, fast cine displacement encoding with stimulated echoes (DENSE), and fast strain encoding (fast-SENC), but their use is limited by prolonged acquisition time and restricted availability [1,4]. Currently, analysis of standard cine imaging represents the most convenient option to assess myocardial strain without the need of acquiring extra sequences. Indeed, modern cine sequences use breath-hold, electrocardiographic-gated, segmented steady-state free precession (SSFP) to produce images with high reproducibility, excellent myocardium-to-blood contrast and high spatial/temporal resolution. Feature tracking analysis detects anatomical features of interest in the LV subendocardium and subepicardium on routinely acquired SSFP cine images and follows them along the cardiac cycle, similarly to echocardiographic speckle tracking [5].

While most of the deformation imaging techniques are based on the similar principles of detecting and tracking specific patterns within an image, there are intra- and inter-imaging modality inconsistencies limiting the wide clinical applicability of strain [6,7]. In particular, normative data on cardiac deformation by feature tracking CMR are of utmost importance to detect early diseases and should be validated from large cohorts of healthy subjects. 

The aim of this study was to derive reference values of LV and RV strain parameters from a large cohort of healthy volunteers from cine CMR imaging. Moreover, we investigated the association of biventricular strain measures with anthropometric data and biventricular function parameters. 

## 2. Materials and Methods

### 2.1. Subjects’ Recruitment

We prospectively enrolled healthy volunteers from May 2018 to March 2020 among the hospital staff and their relatives and through word of mouth. Inclusion criteria were as follows: normal electrocardiogram (performed immediately before the CMR scan), no history of cardiac diseases or symptoms, no cardiovascular risk factors (diabetes, hypertension, dyslipidemia, overweight/obesity, smoking, family history), no known systemic diseases, and no absolute contraindications to CMR. To rule out cardiovascular risk factors and diseases, a lifestyle questionnaire was used to gather data on the individual’s present health condition, medical history, medication history, and smoking habits. Additionally, the most recent blood test was scrutinized as part of the assessment process.

The enrolment process followed a stratified approach to ensure the presence of 10 participants for both genders in each age decile: 20–30 years, 30–40 years, 40–50 years, 50–60 years, and 60–70 years. If a volunteer was excluded after the CMR scan due to pathological findings, another individual of the same gender and age group was recruited in their place to maintain the intended population of 100 volunteers. 

The study complied with the Declaration of Helsinki and was approved by the Institutional Ethics Committee of Area Vasta Nord Ovest (protocol number 17781, year 2018). All subjects gave written informed consent to the protocol.

### 2.2. CMR Protocol

All CMR exams were performed on a 1.5 T scanner (Signa Artist; GE Healthcare, Milwaukee, MI, USA). A 30-element cardiac phased-array receiver surface coil with breath-holding in end-expiration and ECG triggering was used. 

Initially, scout images were acquired to localize the long and short axis of the heart. SSFP cine images were acquired during 8-s breath holds in the vertical and horizontal long axis (LAX) planes, with subsequent contiguous 8-mm short axis slices (SAX) from the atrio-ventricular plane to the apex. The most apical slice included was the first slice showing no blood pool at end-diastole. The most basal slice included was that which showed a remaining part of the thick myocardium and was below the aortic valve. Typical sequence parameters were as follows: repetition time 3.67 ms, echo time of 1.63 ms, flip angle 45°, and matrix size 192 × 192 pixels. There were 30 phases per cardiac cycle.

### 2.3. Image Analysis

Imaging analysis was performed using cvi42 software, version 5.13.7 (Circle Cardiovascular Imaging Inc., Calgary, AB, Canada). 

**Figure 1:**
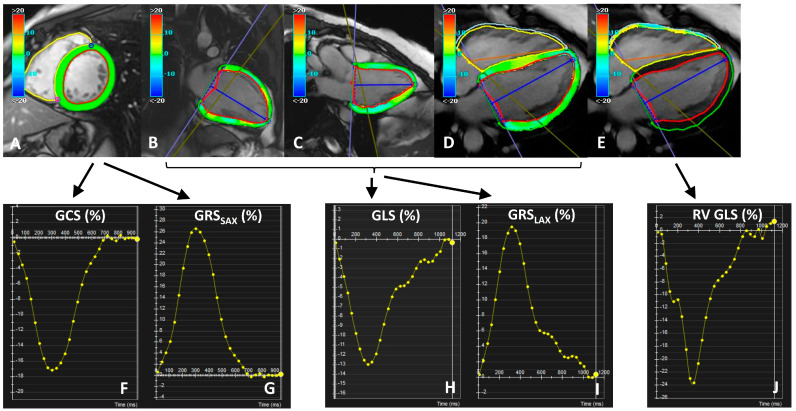
Strain analysis, showing a short axis (**A**), two-chamber (**B**), three-chamber (**C**), and four-chamber (**D**,**E**) view with left-ventricular radial strain (**A**), longitudinal strain (**B**–**D**), and right-ventricular longitudinal strain (**E**) overlay. Please note that the contours were drawn in diastole and systole, and then automatedly propagated and checked throughout all other cardiac phases; papillary muscles and trabeculae were excluded to track the same myocardial structures during the cardiac cycle. GCS (**F**) and GRS_SAX_ (**G**) were calculated from the short-axis cine stack, while GLS (**H**) and GRS_LAX_ (**I**) were calculated from the long axis views. Right-ventricular GLS (**J**) was calculated from the four-chamber cine view.

Feature-tracking analysis was performed by a single operator (R.C.) (Figure 1). For LV strain assessment, short-axis and long-axis cine images were used. LV endocardial and epicardial borders were manually traced on the end-diastolic and and-systolic frames excluding trabeculae, papillary muscles, pericardium, and epicardial fat. For each subject, the end-diastolic and end-systolic phases had to be identical in all SAX and LAX acquisitions. The contours were then automatically propagated (tracked) through the cardiac cycle by matching individual patterns representing anatomical structures. Strain values were calculated by analyzing the relative displacement of the features with respect to the initially defined borders. Tracking quality was checked visually and, if necessary, borders were manually adjusted. For the LV, three-directional myocardial strains were derived: global longitudinal (GLS), radial (GRS), and circumferential (GCS). Global strain was automatically calculated as the average of peak segmental strain of the entire LV. Circumferential and radial_SAX_ strain parameters were derived from the short-axis stack while longitudinal and radial_LAX_ strain parameters were assessed in three LAX views. Due to the high longitudinal displacement of the RV free wall, for the RV, only the GLS was measured, by defining the endocardial and epicardial contours in the four-chamber LAX view; neither RV GRS nor RV GCS were calculated, because of the inherently high through-plane motion of the RV in the SAX views.

Segmental peak strains were also derived for the LV, in accordance with the standardized American Heart Association 17-segment model, with omission of the apex (segment 17) [8]. For SAX strain analysis, basal, mid-ventricular, and apical slices were selected. The basal slice was that still showing a complete circumference of myocardium throughout the entire cardiac cycle while the apical slice was that still showing LV cavity at systole. In each slice, the anterior insertion of the right ventricle was manually defined and used to define the segments (six in basal and mid-ventricular slices and four in apical slice). For further statistical analysis, segments were grouped according to level (basal: segments 1–6; mid-ventricular: segments 7–12; apical: segments 12–16). 

The quantification of biventricular function parameters from SSFP cine images was performed by expert cardiologists (A.B., G.T., C.G., >15 years of experience in CMR) and was based on the manual recognition of the endocardial and epicardial LV contours in end-diastolic and end-systolic phases in each slice [9]. The papillary muscles were delineated and were considered myocardial mass rather than part of the blood pool. End-diastolic volume (EDV) and end-systolic volume (ESV) were identified, respectively, by the global maximum and minimum LV cavity volume, without the need for a geometric assumption of the ventricle shape. The ejection fraction (EF) was given by the ratio between the stroke volume (difference between EDV and ESV) and the EDV. The LV mass was obtained by multiplying the volume of the myocardium by its specific weight (1.05 g/cm^3^). Biventricular volumes and LV mass were indexed to the body surface area (BSA), derived using the variation of the Dubois and Dubois formula [10].

### 2.4. Statistical Analysis

All data were analyzed using SPSS version 27.0 (IBM Corp., Armonk, NY, USA) and MedCalc^®^ version 19.8 (MedCalc Software, Ostend, Belgium) statistical packages. 

The normality of distribution of the parameters was assessed by using the Kolmogorov–Smirnov test or the Shapiro–Wilk test for a sample size ≤ 50. Continuous variables were described as the mean ± standard deviation (SD), and categorical variables were described as frequencies and percentages. 

The comparison between two groups was made using the independent-samples *t*-test for continuous values with normal distribution while the Wilcoxon rank sum test was applied for continuous values with a non-normal distribution. The χ^2^ testing was performed for non-continuous variables.

Correlation analysis was performed using Pearson’s test or Spearman’s test where appropriate. 

Univariate and stepwise multivariate regression analyses were performed to identify determinants of strain measures. Multivariate backward regression was performed using only variables with *p* < 0.10 in univariate regression analyses. To assess the collinearity of variables in the multivariate model, the variance inflation factor (VIF) and tolerance statistic were utilized. A VIF > 5 and/or a tolerance statistic < 0.20 were considered indicative of inflated collinearity.

On the basis of the distribution (normal or not), the lower and upper limits of normal for strain values were calculated on original or log-transformed data as the mean ± 2 SD.

One-way repeated-measures ANOVA or the Friedman test were used to evaluate if there was a significant difference among strain measurements in different segments and slices. The Bonferroni adjustment was applied to account for multiple pairwise comparisons.

A two-tailed *p* < 0.05 was considered statistically significant.

### 2.5. Reproducibility Analysis

Data related to 50 subjects were randomly extracted from the entire dataset and were blindly re-analyzed by the same operator after at least 3 weeks to evaluate the intra-observer agreement and by a different operator (A.B.) to evaluate the inter-observer agreement.

The coefficient of variation (CoV) was obtained as the ratio of the SD of the half mean square of the differences between the repeated values, to the general mean. A CoV < 10% was considered good. The intraclass correlation coefficient (ICC) was obtained from a two-way random effects model with measures of absolute agreement. An ICC ≥ 0.75 was considered excellent, between 0.40 and 0.75 was considered good, and <0.40 was considered unsatisfactory. The agreement between the measurements was determined using the Bland–Altman technique, plotting the difference versus the average of the variables. Bias was the mean of the difference between two datasets and agreement was the mean ±1.96 SDs.

## 3. Results

### 3.1. Study Population

The 100 subjects composing the final study population were all of Caucasian ethnicity. As per inclusion criteria, all volunteers had a normal CMR scan (normal biventricular volumes and ejection fractions, absence of crypts, normal wall motion, and no signs of inflammation or fatty infiltration). Physiological and CMR characteristics of participants are listed in Table 1. Age was comparable between males and females, but males exhibited higher BSA and biventricular volumes and LV mass indexed by BSA, and lower biventricular ejection fractions.

### 3.2. Physiological Correlates of Global Strain Measures

Peak global strains are shown in Table 1. 

LV GRS derived from SAX cines was significantly lower than LV GRS derived from LAX cines (*p* < 0.001), and their correlation was moderate (R = 0.56; *p* < 0.001).

LV GCS showed a very strong significant correlation with LV GRS_SAX_ (R = −0.99; *p* < 0.001) and only a moderate correlation with LV GRS_LAX_ (R = −0.54; *p* < 0.001) and LV GLS (R = 0.55; *p* < 0.001). A very strong association between LV GLS and GRS_LAX_ was detected (R = −0.96; *p* < 0.001). RV GLS exhibited a significant but weak correlation with LV GCS (R = 0.23; *p* = 0.020), LV GRS_SAX_ (R = −0.21; *p* = 0.032), LV GRS_LAX_ (R = −0.36; *p* < 0.001), and LV GLS (R = 0.383; *p* < 0.001). 

There was a significant sex difference in all global LV strain values, as well as in the RV GLS, with a greater deformation among females (Table 1). 

The association of strain measures with physiological parameters is summarized in Table 2. All global LV strains were independent from age and heart rate (HR) but were influenced by BSA. RV GLS was not associated with age, HR, or BSA.

For each type of global strain (dependent variable), the following potential independent variables were tested in univariate regression models: gender, age, BSA, and HR. According to the stepwise multivariate regression analysis, gender emerged as the only significant predictor of all strain measures (Table 3). No variable was excluded from the multivariable models due to excessive collinearity.

### 3.3. Correlation between Strain and Other Measures of Systolic Function

Table 2 shows the association of strain measures with biventricular functional parameters. Both LV GCS and LV GRS_SAX_ were related with LV end-diastolic volume index (EDVI), while no global LV strain parameter was related to LV mass index. A significant association was detected between all LV global strain measures and the LV EF. The global LV strain measures derived from SAX analysis exhibited a significant association with the RV EF. RV GLS was significantly associated with biventricular EDVI but was not correlated with LV or RV EF. 

### 3.4. Reproducibility Results

Table 4 shows the intra-observer and inter-observer reproducibility data for global strain measures. Among global LV strain parameters, GLS was that with the best intra- and inter-operator reproducibility in Bland–Altman analyses. The ICC was excellent and the CoV was <10% for all global strain measures. 

### 3.5. Reference Ranges for Global Strain Values

Lower and upper limits of normal for all global LV strain measures and for the RV strain are shown in Table 5. The distribution was normal for all global strain measures, with the exclusion of the LV GRS_SAX_ which was therefore log-transformed. Due to the gender dependence, gender-specific reference values were developed.

### 3.6. Segmental Strain Values

**Figure 2:**
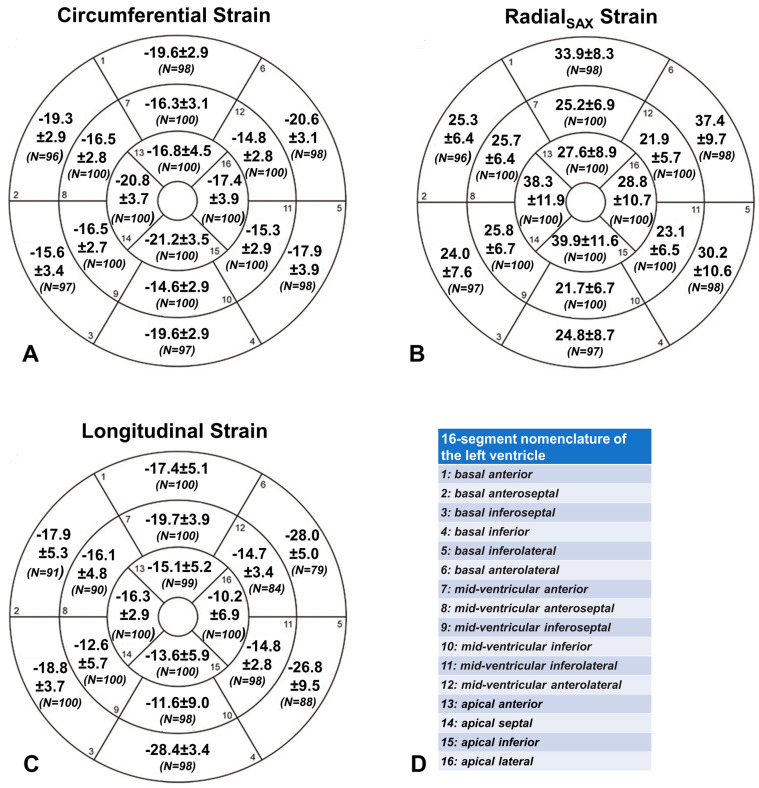
Bullseye plots of segmental circumferential (**A**), radial (**B**), and longitudinal (**C**) strain values [%]. All values are expressed as the mean ± standard deviation. In round brackets, the number (N) of available segments is reported. The 16-segment nomenclature of the left ventricle is summarized in (**D**).

A total of 1600 segments (16 segments × 100 healthy subjects) were evaluated for circumferential, radial, and longitudinal strain. Of the segments acquired in short-axis views, 1584 (99.0%) were assessable. Of the segments obtained in long-axis views, 1525 (95.3%) were interpretable. Figure 2 summarizes the LV segmental strain values. 

**Figure 3:**
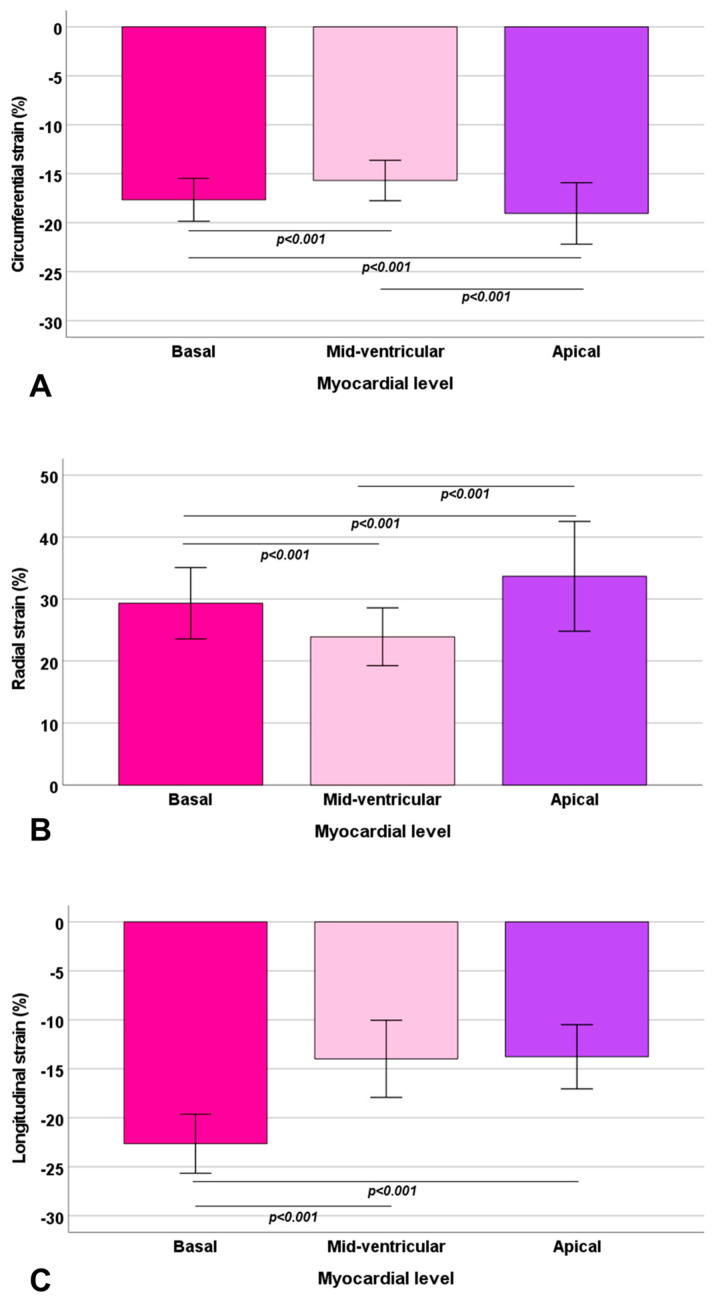
Mean circumferential (**A**), radial (**B**), and longitudinal (**C**) strain values in the three slices. The bars in the boxes represent the standard deviation. The significant differences (*p* < 0.05) between two slices, and the correspondent *p*-values are indicated.

There were significant regional differences in circumferential strain measures (*p* < 0.0001), with the lowest mean value in the mid-ventricular inferior segment (−14.6% ± 2.9%) and the highest in the apical inferior segment (−21.2% ± 3.5%). Significantly larger circumferential strain values were observed at the apical level compared to both basal (−19.1% ± 3.1% vs. −17.7% ± 2.2%; *p* < 0.001) and mid-ventricular level (−19.1% ± 3.1% vs. −15.6% ± 2.0%; *p* < 0.001), and at the mid-ventricular level compared to the basal level (−17.7% ± 2.2% vs. −15.6% ± 2.0%; *p* < 0.001) (Figure 3A).

There were significant regional differences in radial_SAX_ strain measures (*p* < 0.0001), with the lowest mean value in the mid-ventricular inferior segment (21.7% ± 6.7%) and the highest in the apical inferior segment (39.9% ± 11.6%). Significantly larger radial strain values were observed at the apical level compared to both basal (33.9% ± 8.8% vs. 29.3% ± 5.8%; *p* < 0.001) and mid-ventricular level (33.9% ± 8.8% vs. 23.8% ± 4.7%; *p* < 0.001), and at the basal level compared to the mid-ventricular level (29.3% ± 5.8% vs. 23.8% ± 4.7%; *p* < 0.001) (Figure 3B). Regional values of radial_LAX_ strain were also calculated, but they were not included in the final analysis because they displayed very dispersed values with little correlation with radial_SAX_ strain, likely related to the imperfect alignment of the two-chamber, three-chamber, and four-chamber views in tracking segmental deformation values. 

There were significant regional differences in longitudinal strain measures (*p* < 0.0001), with the lowest mean value in the apical lateral segment (−10.2% ± 6.9%) and the highest in the basal inferior segment (−28.4% ± 3.4%). Mean longitudinal strain was significantly higher at the basal level compared to both the mid-ventricular (−22.6% ± 3.0% vs. −13.9% ± 3.9%; *p* < 0.001) and the apical level (−22.6% ± 3.0% vs. −13.8% ± 3.3%; *p* < 0.001), while no difference was detected between the longitudinal strain at the mid-ventricular and apical levels (*p* = 0.87) (Figure 3C).

## 4. Discussion

This article derived reference values of LV GCS, LV GRS_SAX_, LV GRS_LAX_, LV GLS, and RV GLS. Women presented higher global LV and RV strain values than men; all strain values presented a weak relationship with body surface area, while there was no association with age or heart rate. A significant association was detected between all LV global strain measures and LV ejection fraction, while RV GLS was correlated to RV end-diastolic volume. The ICC was excellent and the CoV was <10% for all global strain measures, with LV GLS showing the best intra- and inter-operator reproducibility. Regional values were also calculated for the LV, with circumferential and radial strain values resulting higher at the apical level, while longitudinal strain values were higher at the basal level.

Our results are in agreement with previous results, even though the reference values found from different studies do not completely overlap (Table 6). Of note, our article is the only one to calculate radial strain from both long-axis and short-axis planes, while, in almost all previous studies, horizontal long-axis cines were tracked to derive longitudinal strain, while short-axis cines were used to derive circumferential and radial strain. Moreover, the gender differences in myocardial strain are in agreement with previous studies [11,12,13], while the relationship between age and strain values has shown conflicting results. In particular, differently from our results, several previous studies reported an age-related increase in LV GCS [11,12,13], LV GRS [12,13,14], and all RV strains [13]. On the other hand, in children, all global LV strains showed a significant parabolic relation to age and an even stronger one to BSA, but no gender differences [15]. In a recent meta-analysis including 44 studies with a total of 3359 healthy subjects, the pooled means of LV-GLS and RV-GLS were very close to our findings, while LV-GRS and LV-GCS were slightly higher. In the same meta-analysis, magnetic field strength and feature tracking vendor emerged as significant confounders contributing to heterogeneity of global strain values, whereas sex, age, and MR vendor had no effect [16]. 

Overall, our analysis focused on the most commonly used strain parameters, i.e., GCS, GRS, and GLS for the LV and GLS for the RV. Previous studies have demonstrated the prognostic role of these global strain values, particularly of LV GLS, across almost all cardiovascular diseases [22,23,24,25,26,27,28,29]. Currently, feature tracking CMR has become a feasible and clinically useful tool for myocardial strain calculation of all cardiac chambers in ischemic and nonischemic heart diseases [30]. 

On the other hand, LV segmental wall motion analysis is important for clinical decision making in cardiac diseases, but the finding that segmental deformation assessment with feature tracking is far less reliable than global strain estimation is in line with previous results [31,32]. Segmental strain values presented a much higher variability than global values; thus, the derivation of segmental reference range still needs further larger studies before it can be used in clinical practice. Feature tracking results are highly dependent on reader experience and on accurate detection of epicardial and endocardial contours [33]; even when particular care is taken to endocardial and epicardial contouring throughout the cardiac cycle, this inherent limitation might explain the inadequate accuracy of segmental analysis. In our analysis, both end-diastolic and end-systolic contours were manually drawn, to minimize inaccuracies of automated contour propagation that may overestimate inward motion of the endocardial contour when the blood spaces between the trabeculae close during systole. Moreover, manual correction of automated contouring between end-systole and end-diastole was limited to correct gross errors, in line with previous studies, in order to minimize interobserver variability. A significant improvement in strain measurement may come from 3D feature tracking CMR; even though its reference values are slightly different (i.e., consistently lower) compared to 2D measures, 3D strain analysis displays an improved reproducibility, particularly for radial strain, which is perhaps most sensitive to through plane feature loss [19].

### Limitations

Several limitations should be acknowledged in our study. 

First, only biventricular systolic strain was analyzed, while other parameters (systolic strain rate, early and late diastolic strain rate) and other cardiac chambers (left and right atrium) were not considered in this analysis. On the other hand, the lower temporal resolution of CMR compared with echocardiography raises concerns over the theoretical accuracy of any CMR-based deformation algorithm in assessing systolic and diastolic strain rates; in particular, strain rate values have been found to correlate with the number of cardiac phases per cardiac cycle [12]. 

Second, despite extra care being taken to track the endocardial and epicardial contours throughout the cardiac cycle, we found a limited reproducibility of regional strain analysis, in line with previous studies [31,32], making it impossible to derive normative strain values on a segmental basis. In particular, radial_LAX_ strain presented very dispersed segmental values with little correlation with radial_SAX_ strain, likely related to the imperfect alignment of the two-chamber, three-chamber, and four-chamber views in tracking segmental deformation values; again, this is in line with the previous literature, which calculated radial strain from short axis only. 

A third limitation is that, for the RV, only GLS was measured, while GCS, GRS, and regional values were not measured. 

Lastly, all subjects involved in this study were Caucasian, limiting generalizability to other races.

## 5. Conclusions

The assessment of cardiac deformation by CMR with a post-process feature tracking analysis of common cine SSFP images is feasible and reproducible. Several different gender-specific reference global values were derived for the LV and RV, while regional values resulted quite interspersed with limited reproducibility. 

Further larger analyses are needed for the assessment of normative values of biventricular strain rate, of atrial strain and strain rate, and of segmental biventricular values.

## Figures and Tables

**Table 1:** Physiological and CMR characteristics of healthy volunteers.

	All (N = 100)	Males (N = 50)	Females (N = 50)	*p*-Value
***Age*** **(years)**	44.7 ± 14.2	44.6 ± 14.4	44.7 ± 14.1	0.89
**Caucasian race, N (%)**	100 (100)	50 (100)	50 (100)	-
**Body surface area (m^2^)**	1.8 ± 0.2	1.9 ± 0.2	1.7 ± 1.5	<0.001
**Heart Rate (bpm)**	65.6 ± 10.0	64.0 ± 11.8	67.1 ± 7.7	0.13
**LV end-diastolic volume index (mL/m^2^)**	74.8 ± 12.7	79.5 ± 12.4	70.1 ± 11.3	<0.001
**LV end-systolic volume index (mL/m^2^)**	27.5 ± 7.3	30.5 ± 7.1	24.5 ± 6.3	<0.001
**LV stroke volume index (mL/m^2^)**	47.4 ± 8.4	49.1 ± 8.1	45.7 ± 8.6	0.027
**LV mass index (g/m^2^)**	54.9 ± 12.5	60.3 ± 9.9	49.5 ± 12.6	<0.001
**LV ejection fraction (%)**	63.5 ± 6.1	61.8 ± 5.4	65.1 ± 6.4	0.012
**RV end-diastolic volume index (mL/m^2^)**	74.0 ± 14.3	80.6 ± 13.4	67.4 ± 11.9	<0.001
**RV end-systolic volume index (mL/m^2^)**	28.9 ± 8.3	32.7 ± 7.5	25.1 ± 7.4	<0.001
**RV stroke volume index (mL/m^2^)**	45.4 ± 8.7	47.9 ± 8.7	42.8 ± 8.0	0.003
**RV ejection fraction (%)**	61.8 ± 5.5	59.9 ± 4.9	63.5 ± 5.5	0.001
**LV GCS (%)**	−16.7 ± 2.1	−16.0 ± 1.9	−17.4 ± 2.1	0.001
**LV GRS_SAX_ (%)**	26.4 ± 5.1	24.8 ± 4.5	27.9 ± 5.2	0.001
**LV GRS_LAX_ (%)**	31.1 ± 5.2	28.9 ± 4.4	33.3 ± 5.1	<0.001
**LV GLS (%)**	−17.7 ± 1.9	−16.9 ± 1.7	−18.5 ± 1.7	<0.001
**RV GLS (%)**	−23.9 ± 4.1	−23.0 ± 3.6	−24.7 ± 4.3	0.036

Continuous variables are expressed as the mean ± standard deviation and categorical variables are expressed as frequencies (%). The *p*-values refer to the comparison between males and females and are considered statistically significant when below 0.05. N = number; LV = left ventricular; RV = right ventricular; GCS = global circumferential strain; GRS= global radial strain; SAX = short axis; LAX = long axis; GLS = global longitudinal strain.

**Table 2:** Association of global strain parameters with physiological and CMR parameters.

	Age	BSA	HR	LV EDVI	LV Mass Index	LV EF	RV EDVI	RV EF
**LV GCS (%)**	R = −0.06; *p* = 0.53	**R = 0.21; *p* = 0.040**	R = −0.09; *p* = 0.383	**R = 0.24; *p* = 0.015**	R = 0.16; *p* = 0.11	**R = −0.73; *p* < 0.001**	R = 0.12; *p* = 0.25	**R = −0.50; *p* < 0.001**
**LV GRS_SAX_ (%)**	R = 0.06; *p* = 0.57	**R = −0.28; *p* = 0.005**	R = 0.07; *p* = 0.499	**R = −0.25; *p* = 0.014**	R = −0.18; *p* = 0.09	**R = 0.72; *p* < 0.001**	R = −0.15; *p* = 0.14	**R = 0.49; *p* < 0.001**
**LV GRS_LAX_ (%)**	R = −0.18; *p* = 0.07	**R = −0.35; *p* < 0.001**	R = −0.05; *p* = 0.617	R = −0.08; *p* = 0.43	R = −0.14; *p* = 0.18	**R = 0.35; *p* < 0.001**	R = −0.01; *p* = 0.89	R = −0.16; *p* = 0.12
**LV GLS (%)**	R = 0.19; *p* = 0.06	**R = 0.34; *p* < 0.001**	R = 0.06; *p* = 0.569	R = 0.12; *p* = 0.26	R = 0.12; *p* = 0.24	**R = −0.37; *p* < 0.001**	R = 0.01; *p* = 0.98	R = −0.12; *p* = 0.26
**RV GLS (%)**	R = −0.09; *p* = 0.37	R = 0.11; *p* = 0.27	R = −0.13; *p* = 0.202	**R = 0.23;** ***p* = 0.022**	R = −0.13; *p* = 0.21	R = 0.14; *p* = 0.18	**R = 0.22;** ***p* = 0.032**	R = −0.11; *p* = 0.27

Pearson’s or Spearman’s correlation coefficient (R) and significance level (*p*-value) are presented. Data in bold indicate statistically significant associations (*p* < 0.05). BSA = body surface area; HR = heart rate; LV = left ventricular; EDVI = end-diastolic volume index; EF = ejection fraction; RV = right ventricular; GCS = global circumferential strain; GRS= global radial strain; SAX = short axis; LAX = long axis; GLS = global longitudinal strain.

**Table 3:** Linear regression analyses of strain measures.

Dependent Variable	Independent Predictors	Univariate Analysis		Multivariate Analysis	
		Beta Coefficient (95% CI)	*p*-Value	Beta Coefficient (95% CI)	*p*-Value
**LV GCS (%)**	Sex (M = 1; F = 2)	−1.4 (−2.2; −0.57)	0.001	−1.4 (−2.2; −0.57)	0.001
	Age	−0.01 (−0.03; 0.02)	0.52		
	BSA	1.9 (0.09; 3.9)	0.040		
	HR	−0.02 (−0.06; 0.02)	0.38		
**LV GRS_SAX_ (%)**	Sex (M = 1; F = 2)	3.2 (1.3; 5.2)	0.001	3.2 (1.3; 5.2)	0.001
	Age	0.02 (−0.05; 0.09)	0.54		
	BSA	−4.7 (−9.3; −0.19)	0.042		
	HR	0.04 (−0.06; 0.14)	0.41		
**LV GRS_LAX_ (%)**	Sex (M = 1; F = 2)	4.4 (2.5; 6.3)	<0.001	4.4 (2.5; 6.3)	<0.001
	Age	−0.05 (−0.12; 0.02)	0.16		
	BSA	−7.9 (−12.2; −3.7)	<0.001		
	HR	−0.03 (−0.13; 0.08)	0.62		
**LV GLS (%)**	Sex (M = 1; F = 2)	−1.5 (−2.2; −0.82)	<0.001	−1.5 (−2.2; −0.82)	<0.001
	Age	0.03 (−0.00; 0.05)	0.06		
	BSA	2.8 (1.2; 4.3)	0.001		
	HR	0.01 (−0.03; 0.05)	0.57		
**RV GLS (%)**	Sex (M = 1; F = 2)	−1.7 (−3.3; −0.11)	0.036	−1.7 (−3.3; −0.11)	0.036
	Age	−0.03 (−0.08; 0.03)	0.37		
	BSA	2.6 (−1.0; 6.2)	0.16		
	HR	−0.05 (−0.13; 0.03)	0.23		

Only those variables with a *p* < 0.10 in the univariate regression analyses are included in the multivariate backward stepwise regression analysis. A *p*-values < 0.05 is considered statistically significant. CI = confidence intervals; LV = left ventricular; GCS = global circumferential strain; GRS = global radial strain; SAX = short axis; LAX = long axis; GLS = global longitudinal strain; RV = right ventricular; M = male; F = female; BSA = body surface area; HR = heart rate.

**Table 4:** Intra- and inter-observer reproducibility of global strain measures.

Measure	INTRA-OPERATOR				INTRA-OPERATOR			
	Bland–Altman Analysis		CoV (%)	ICC [95% CI]	Bland–Altman Analysis		CoV (%)	ICC [95% CI]
	Bias (%)	Limits (%)			Bias (%)	Limits (%)		
**LV GCS (%)**	−0.94	−2.1 to 0.18;	4.4	0.95 [0.63; 0.99]	−0.28	−1.5 to 0.91	2.8	0.98 [0.96; 0.99]
**LV GRS_SAX_ (%)**	1.9	−2.6 to 6.5	7.3	0.95 [0.69; 0.99]	0.78	−2.6 to 4.2	4.9	0.97 [0.94; 0.99]
**LV GRS_LAX_ (%)**	1.8	−3.6 to 7.3	5.5	0.83 [0.40; 0.95]	−0.09	−4.9 to 4.7	5.7	0.93 [0.87; 0.96]
**LV GLS (%)**	−0.65	−2.6 to 1.3	3.9	0.85 [0.46; 0.95]	0.09	−1.7 to 1.9	3.7	0.93 [0.88; 0.97]
**RV GLS (%)**	−0.04	−3.8 to 3.7	5.2	0.88 [0.64; 0.96]	1.2	−3.7 to 6.1	7.7	0.83 [0.61; 0.92]

CoV = coefficient of variation; ICC = intraclass correlation coefficient; CI = confidence interval; LV = left ventricular; GCS = global circumferential strain; GRS = global radial strain; SAX = short axis; LAX = long axis; GLS = global longitudinal strain; RV = right ventricular.

**Table 5:** Gender-specific reference ranges for global strain values.

	Males	Females
**LV GCS** **(%)**	−12.3 to −19.8	−13.2 to −21.6
**LV GRS_SAX_** **(%)**	17.3 to 34.1	17.5 to 38.4
**LV GRS_LAX_** **(%)**	20.1 to 37.7	23.2 to 43.4
**LV GLS** **(%)**	−13.7 to −20.3	−14.9 to −21.9
**RV GLS** **(%)**	−15.8 to −32.5	−16.1 to −33.4

LV = left ventricular; GCS = global circumferential strain; GRS = global radial strain; SAX = short axis; LAX = long axis; GLS = global longitudinal strain; RV = right ventricular.

**Table 6:** Reference strain values from CMR studies.

Study	Year	Sample Size (N)	LV GCS (%)	LV GRS (%)	LV GLS (%)	RV GLS (%)
Augustine et al. [17]	2013	145	−21 ± 3	25 ± 6	−19 ± 3	n.a.
Taylor et al. [11]	2015	100	−26.1 ± 3.8	39.8 ± 8.3	−21.3 ± 4.8	n.a.
Andre et al. [14]	2015	150	−21.3 ± 3.3	36.3 ± 8.7	−21.6 ± 3.2	n.a.
Troung et al. [18]	2017	50	n.a.	n.a.	n.a	−22.11 ± 3.51
Liu et al. [19]	2018	100	−20.9 ± 3.6	46.6 ± 15.4	−19.8 ± 2.9	n.a.
Peng et al. [20]	2018	150	−24.3 ± 3.1	79 ± 19.4	−22.4 ± 2.9	−29.3 ± 6.0
Vo et al. [21]	2018	659 (metanalysis from 18 studies)	−23.0	−34.1	−20.1	−21.8
Weise Valdés et al. [12]	2021	181	−19.2 ± 2.1	34.2 ± 6.1	−16.9 ± 1.8	n.a.
Li et al. [13]	2022	566	−19.6 ± 2.1	34.5 ± 6.3	−16.6 ± 2.1	−21.2 ± 5.0
Yantg et al. [16]	2023	3359 (metanalysis from 44 studies)	−21.4%	43.7%	−18.4%	−24.0%
Barison et al.	2023	100	−16.7 ± 2.1	26.4 ± 5.1	−17.7 ± 1.9	−23.9 ± 4.1

Strain values are expressed as the mean or as the mean ± standard deviation. N = number; LV = left ventricular; GCS = global circumferential strain; GRS= global radial strain; GLS = global longitudinal strain; RV = right ventricular; n.a. = not assessed.

## Data Availability

The data underlying this article cannot be shared publicly due to privacy reasons. The data will be shared on reasonable request to the corresponding author.
